# Evaluation of China’s low-carbon city pilot policy: Evidence from 210 prefecture-level cities

**DOI:** 10.1371/journal.pone.0258405

**Published:** 2021-10-07

**Authors:** Shuang Zhou, Chaobo Zhou

**Affiliations:** Economics and Management School, Wuhan University, Wuhan, China; Northeastern University (Shenyang China), CHINA

## Abstract

As the largest carbon dioxide emitter, China is working towards the direction of a green economy. As an irreplaceable part of establishing a green economy, the low-carbon city pilot (LCCP) policy is implemented in many large cities in China, and the scope of implementation will be further expanded. However, to date, there has been an absence of empirical studies basing on prefecture-level cities about the evaluation of China’s LCCP policy. Evaluating and optimizing the LCCP policy is constructive to achieve the goal of China’s green economic transition. In this paper, we evaluated the effect of the LCCP policy on China’s low-carbon economic transition by using the difference-in-difference (DID) approach which can effectively alleviate endogenous problems and better evaluate this effect and the panel data of 210 prefecture-level cities in China from 2008 to 2016. The empirical analysis revealed that the LCCP policy inhibited China’s low-carbon economic transition in general. Specifically, the policy worked well in the eastern region but failed in the central region and western region by studying the regional heterogeneity and influence mechanism. The reason is that the LCCP policy can stimulate low-carbon innovation with the help of innovation offset effects in the eastern region, but it failed to do so in the central region and western region. In addition, this paper analyzed the performance of three types of policy tools adopted by local governments to implement the policy, we found that market-economic tools are valuable to improving the low-carbon economic transition in pilot areas, but command-mandatory tools and voluntary tools have failed to achieve the expected objectives. The research results of this article can provide policy recommendations for optimizing the low-carbon policy and provide a reference for countries that are determined to develop a green economy.

## Introduction

With the process of industrialization, the emission of greenhouse gases has become one of the global challenges [1]. Since the implementation of the reform and opening-up policy, China’s economy has been developing rapidly. One of the costs of excessive development is that voluminous carbon dioxide and concomitant dust particles are produced, which not only raised the temperature but also polluted the natural environment. Scholars in many fields have shown keen concern about temperature and its influence. For example, they observed the urban heat island (UHI) phenomenon, He, Zhao [2] and Zhao, He [3] used the largest city in the northeast of China, Shenyang City, as a case to examined and analyzed environmental temperatures and land surface temperature (LST), and have reached some significant conclusions. To cease the further rise in global temperature, many countries have been working hard to reduce greenhouse gas emissions, including China. The participating countries of the Paris Agreement unanimously agreed that by the end of this century efforts should be made to limit the increase in global average temperature within 1.5°C and at most 2°C [4]. Besides, the Chinese government promised that the intensity of carbon emissions by 2020 would be reduced by 40% to 45% compared with 2005 to alleviate climate change at the United Nations Climate Change Conference in Copenhagen [5]. To save energy and protect the environment, China’s National Development and Reform Commission (NDRC) designated three groups of pilot regions in 2010, 2012, and 2017 to execute a series of measures that target low-carbon transition for the entire city including low-carbon production and low-carbon consumption. These measures implemented in the pilot regions constitute the LCCP policy.

Because of the shortage of resources and the deterioration of the environment, a sustainable development model is imminent globally. To develop a low-carbon economy is to build a benign and sustainable energy ecosystem. The term "low-carbon economy" originated from the energy white paper of the British government in 2003, which represents a sustainable and low-carbon economic growth pattern. The core idea of low-carbon transition is to shift the economic development model from low high-carbon emissions type to low-carbon emissions type. Facing China’s objective demand for green development and the LCCP policy designed to meet this demand by China’s NDRC, we are engrossed in the following questions. Is the LCCP policy beneficial to China’s sustainable economic transition? If it is, what are the influence channels? Is there regional heterogeneity in the influence of the LCCP policy on China’s sustainable development? And what is the performance of the various policy tools used by the pilot cities? Research on these questions can provide policy recommendations for the policymakers to perfect China’s LCCP policy and provide practical experience for the world, especially developing countries and regions.

As a developing country and the largest carbon emitter, the influence of China’s LCCP policy on industrial structure, energy structure, and emission reduction has attracted extensive attention domestically and abroad since the LCCP policy implementation. The existing research that evaluates the policy can roughly fall into two categories. The first is that the LCCP policy is beneficial to China’s low-carbon transition. For instance, Lu, Wang [6] found that China’s LCCP policy benefited the upgrading of industrial structure in pilot regions. Meanwhile, the results indicated that it had a positive spatial spillover effect. Song, Zhao [7] explored that the LCCP policy can improve energy efficiency in pilot regions. Zhang [8] elaborated that China’s LCCP policy can curb carbon emissions by reducing electricity consumption and improving the level of technological innovation. Song, Sun [9] regarded that China’s LCCP policy can diminish urban air pollution by reducing enterprise carbon emissions and upgrading the industrial structure. Song, Qin [10] believed that policy innovation is encouraged by the LCCP policy through coordination mechanism and financing mechanism. Wang, Chen [11]explored that the LCCP policy can effectively improve the total factor productivity of local enterprises.

The other is that the LCCP policy is invalid or even adverse to China’s low-carbon transition. The negative comments on this policy mainly come from three perspectives. The first is the design flaws in the formulation of the policy. The policy is short of a clear definition, effective evaluation system, and specific goals, which makes the policy implementation chaotic [12]. In addition, the low level of citizen participation as stakeholders in policy formulation and implementation has led to poor environmental performance in the pilot regions [13]. The last is that the resource allocation under the policy is distorted and inefficient, leading to a Green Paradox [14]. Therefore, the institutional defects may cause the LCCP policy to achieve undesirable results, showing that the policy should be examined and optimized.

The researches about China’s low-carbon transition mainly focus on its definition [15] and measurement indicators. Specifically, the great majority of studies adopted carbon productivity as an indicator to measure the low-carbon economic transition [11]. Under the background of low-carbon development, it is a common challenge for developing countries to achieve the balance between controlling carbon emissions and maintaining economic growth. Technically, the only way to reduce carbon emissions and maintain economic growth is to improve carbon productivity. Therefore, carbon productivity is considered as the core indicator to measure the low-carbon transition. It can measure the level of low-carbon technology of a country or a region in a certain period, and use it to evaluate the carbon cost per unit of economic growth. Therefore, by improving carbon productivity, a country can achieve the goal that lower carbon emissions matching greater economic output occur [16, 17]. In short, the core of constructing a low-carbon economy is to improve carbon productivity. Li, Hu [18] studied the influence of environmental regulations on China’s carbon productivity, and found that environmental regulations can effectively promote China’s carbon productivity. The LCCP policy is one of the environmental regulations, but there are few studies about its influence on carbon productivity.

To sum up, there has been some research evaluating China’s LCCP policy, and they formed opposite opinions on this. However, there are no adequate studies on the effect of the LCCP policy, a market-driven environmental regulation, on China’s export technical sophistication. The reason is that the objects affected by the policy and the data used in the existing literature research are different. These studies have yielded seemingly very different results, exacerbating differences. Additionally, there is a lack of studies on the effect of China’s LCCP policy on China’s low-carbon economic transition, especially on the influence channels, regional heterogeneity, and policy tool performance.

Compared with existing literature, this paper contributes 3 innovations. (1) This paper uses the DID method to calculate the influence of the LCCP policy on China’s economic transition from national and regional perspectives, providing direct empirical evidence for optimizing the policy. (2) In this paper, a mediation variable is found to explore the influence channel of the LCCP policy on China’s low-carbon transition and to further assess the influence channel from the perspective of regional heterogeneity. (3) This paper analyzed the performance of different tools of the policy implementation in pilot regions, helping to improve the effectiveness of the low-carbon policy.

## Policy background and hypothesis

### The LCCP Policy and China’s low-carbon economic transition

The LCCP policy has designated three batches of cities and provinces to deliver low-carbon transition, and the implementation process of this policy is as follows. In 2010, the NDRC implemented the LCCP policy for the first time, which designated five provinces including Guangdong Province, Liaoning Province, Hubei Province, Shanxi Province, and Yunnan Province, and eight cities including Tianjin City, Chongqing City, Shenzhen City, Xiamen City, Hangzhou City, Nanchang City, Guiyang City, and Baoding City as pilot regions. It may be because the Chinese government was uncertain about the effect of this policy in different regions, the first batch of pilot regions were evenly distributed in China. And the eight cities were all provincial capitals, which ensured that there were sufficient resources to implement the policy. In 2012, for expanding the scope of the pilots the NDRC implemented the LCCP policy for the second time, one province, namely Hainan Province, and twenty-eight cities, namely Beijing City, Shanghai City, Shijiazhuang City, Qinhuangdao City, Jincheng City, Hulunbuir City, Jilin City, Daxinganling Area, Suzhou City, Huai’an City, Zhenjiang City, Ningbo City, Wenzhou City, Chizhou City, Nanping City, Jingdezhen City, Ganzhou City, Qingdao City, Jiyuan City, Wuhan City, Guangzhou City, Guilin City, Guangyuan City, Zunyi City, Kunming City, Yan’an City, Jinchang City, and Urumqi City were included in the policy. The implementation of the LCCP policy has been extended to prefecture-level cities. Statistical analysis indicated that this policy has had a significant effect on reducing carbon emissions in some pilot cities. For example, Hangzhou City, Xiamen City, and Shenzhen City have reduced carbon emissions by more than 200,000 tons per year [19, 20]. And, compared with the provincial average level, the carbon emission intensity of Zunyi City, Urumqi City, and Wuhan City decreased by 20.43%, 19.44%, and 19.12%, respectively [21]. As a result, in 2017 the NDRC launched the third batch of pilot cities covering 43 cities, namely Wuhai City, Shenyang City, Dalian City, Chaoyang City, Xunke County, Nanjing City, Changzhou City, Jiaxing City, Jinhua City, Quzhou City, Hefei City, Huaibei City, Huangshan City, Lu’an City, Xuancheng City, Sanming City, Gongqing City, Ji’an City, Fuzhou City, Jinan City, Yantai City, Weifang City, Changsha City, Zhuzhou City, Xiangtan City, Chenzhou City, Zhongshan City, Liuzhou City, Sanya City, Chengdu City, Yuxi City, Pu’er Simao District, Lhasa City, Ankang City, Lanzhou City, Dunhuang City, Xining City, Yinchuan City, Wuzhong City, Changji City, Yining City, Hotan City, and First Division Alar City and two counties, namely Changyang Tujia Autonomous County, Qiongzhong Li, and Miao Autonomous County.

Each implementation of the LCCP policy contains five or six policy objectives that are slightly different, of which three core objectives have not changed. They include formulating low-carbon development plans, calculating and controlling carbon dioxide emissions, and transforming industries from high-carbon to low-carbon. The LCCP policy has been implemented in many cities in China, so it is important to study the relationship between reducing carbon dioxide emissions and economic efficiency in pilot areas. In other words, whether the LCCP policy promotes carbon productivity and China’s low-carbon economic transition is worth studying.

Porter Hypothesis holds that proper environmental regulations can urge enterprises to develop technological innovation, which lowers the production costs and offsets compliance costs [22]. The LCCP policy used administrative methods and tax incentives to stimulate enterprises to develop low-carbon technologies [23]. Additionally, the policy adopted various ways to subsidize related enterprises, such as low-carbon development funds, investment subsidies, loan interest discounts, direct rewards, and project management fee subsidies, to expand their R&D expenditures on low-carbon technology. Technological innovations lead to higher carbon productivity, which not only compensates for the compliance cost but also makes the enterprises generate fewer carbon emissions than those not investing in low-carbon technologies [24]. Additionally, Gong, Liu [25] found that the LCCP policy significantly promoted foreign direct investment. Technological innovation has spillover effects because foreign enterprises possessing advanced technologies spread greener production technologies to host countries to help them to improve their environmental protection levels. In summary, we believe that the LCCP policy can promote low-carbon technological innovation which is beneficial to low-carbon economic transition. Therefore, we propose the first hypothesis:

Hypothesis 1: the LCCP policy can promote low-carbon economic transition by improving the low-carbon innovation capabilities of enterprises in pilot regions.

### Policy tools and enterprise green technology innovation

As mentioned above, the LCCP policy does not have specific quantitative targets, financial supports, and compensation rules, meaning that local governments can freely choose implementation paths and tools. Generally, local governments use three types of policy tools to build low-carbon cities, including command-mandatory tools, market-economic tools, and voluntary tools [26].

Command-mandatory tools used in the LCCP policy mainly include outdated production elimination, emission control standards for motor vehicles, low energy consumption for green buildings, vehicle emission standards. For example, Tianjin City, one of the eight first pilot cities, participated in the National Energy Conservation Plan, so the Tianjin government required 211 local enterprises to save 4.86 million tons of standard coal. Market-economic tools applied by the LCCP policy mainly consist of low-carbon subsidies, preferential interest loans for low-carbon programs, carbon emissions trading, tax incentives. For instance, in 2011 two provinces, namely Hubei Province and Guangdong Province and five municipalities, namely Beijing City, Shanghai City, Tianjin City, Chongqing City, and Shenzhen City conduct the Carbon Emission Trading Pilot Scheme (ETPS). Voluntary tools adopted in the LCCP policy mainly comprise low-carbon transportation programs, low-carbon industrial park programs, carbon monitoring. For example, Tianjin has established a green building certification system and standards. Additionally, Hangzhou City has adopted the low-carbon product certification system by using ISO 14064 and PAS 2050 and encouraged local enterprises to reduce carbon emissions per unit product.

Three policy tools affect the low-carbon economic transition differently. Command-mandatory tools aim to formulate strict emission reduction targets and clear technical standards to limit the pollution emissions of enterprises, which would inevitably increase the operating costs of enterprises in terms of pollution discharge and pollution control. Market-economic tools are relatively flexible, mainly using market mechanisms to provide economic incentives for enterprises’ innovative behavior. Voluntary tools are to arouse enterprises’ environmental awareness and enable them to spontaneously reduce carbon emissions. Among the three tools, the rigid command-mandatory tools are likely to ignore the corporate capability of pollution control. For meeting policy requirements, enterprises must reduce energy consumption and use other alternatives, which leads to higher costs and damages their comparative advantages and profitability. Additionally, strict environmental policies have also caused difficulties for enterprises in management, such as spending more time and energy [27]. As a result, command-mandatory tools failed to evoke the low-carbon economic transition in pilot areas. Voluntary tools are the least restrictive to enterprises, so they may not have a profound impact on the innovation capabilities of enterprises and have limited influence on the low-carbon economic transition in pilot regions [28]. However, flexible market-economic tools not only strongly encourage enterprises to innovate low-carbon technology but also bring innovation compensation to participating enterprises. Therefore, market-economic tools are advantageous to reduce carbon emissions and superior to construct a low-carbon economy. In summary, we propose the second hypothesis:

Hypothesis 2: In the LCCP policy implementation, market-economic tools are constructive on developing low-carbon economic transformation in pilot regions.

## Data and methodology

### Data description

For evaluating the effect of the LCCP policy on China’s low-carbon economic transition, this paper took 286 prefecture-level cities in China from 2008 to 2016 as the original samples. This article selected the second batch of pilot regions as the empirical subjects. On the one hand, the first batch of pilot regions selected provinces as the main body, meaning that the number of pilot regions is relatively small. And the level of economic development of these pilot regions is relatively high, meaning that the research on the first batch of pilot regions is not very representative. On the other hand, the third batch of pilot regions started late, so the policy effects have not yet appeared. By learning from the research of Cheng, Yi [23] and Song, Zhao [7], we used the second batch of pilot cities as the research samples. For eliminating the interference from the first batch of pilot cities, we removed the first batch of pilot cities from the original samples and used the second batch of pilot cities as the experimental group and other non-pilot cities as the control group. Finally, 210 cities were selected as the empirical samples. The data are from *China’s urban statistical yearbook* from 2009 to 2017. For eliminating the impact of price fluctuations, all GDP-related data were adjusted by the GDP deflator to the year 2008.

#### Core variables

The first core variable is the low-carbon economic transition as the explained variable. This article used carbon productivity (*CP*) to measure the level of low-carbon economic transition based on Wang, Chen [11]. Carbon productivity refers to the level of GDP output per unit of carbon dioxide, specifically the ratio of GDP to carbon dioxide emissions. Carbon productivity is the most applied in existing studies to describe the transition of a low-carbon economy [11]. It is a common dilemma for developing countries to keep the balance between ecosystem and economic growth. Working on carbon productivity can make greater economic growth with lower carbon emissions [29]. Therefore, we adopted carbon productivity as an indicator of the economic low-carbon transition.

Although *China’s Urban Statistical Yearbook* does not provide specific data on urban carbon emissions, it does provide consumption of natural gas, liquefied petroleum gas, and electricity. Basing on these indirect data and IPCC (2006) conversion standards, and referring to the practices of Cheng, Yi [23] and Zhang, Deng [30], we calculated the carbon dioxide emissions of each city. For the consideration of heteroscedasticity, this article takes the logarithm of carbon productivity (*lnCP*).

The second core variable is low-carbon innovation (*LCI*) as a mediation variable. Patent authorization standards are objective and stable, so the number of patents can reflect the level of innovation [31]. The patients classified as Y02 are green technologies and applications for mitigating or adapting to climate change in the patent classification catalog jointly issued by the European Patent Office and the US Patent Office [32]. This article regarded the patents classified as Y02B, Y02C, Y02D, Y02E, Y02P, Y02T, and Y02W as low-carbon innovation patients, and adopted their total number as a low-carbon innovation indicator for each city [33]. For the consideration of heteroscedasticity, this article takes the logarithm of low-carbon innovation (*lnlci*).

#### Control variables

We also selected other indicators that may influence the pilot policy on the low-carbon economic transition, including industrial structure, foreign direct investment, total population, infrastructure, research, and development intensity, and economic development level [11, 34–36].

Specifically, industrial structure (*IS*) is the ratio of the added value of the secondary industry to the added value of the tertiary industry, Bu, Qiao [28] used the Logarithmic Mean Divisia Index (LMDI) method to calculate China’s carbon dioxide emissions which are decomposed into economic aggregate effects, industrial structure effects, and energy intensity effects from 1980 to 2010 and emphasized that changes in industrial structure have a significant effect on carbon productivity. Therefore, we use the industrial structure as a control variable.

Foreign direct investment (*FDI*) is the ratio of the city’s foreign direct investment to the regional GDP. [8] found that technological innovation has spillover effects because foreign enterprises possessing progressive technologies spread greener production technologies to host countries to assist them in raising their environmental protection levels. Therefore, we use FDI as a control variable.

Total population (*POP*) is the number of permanent residents in the city. [35] believe that the influence of population size on carbon emissions cannot be ignored, and population growth leads to an increase in total carbon emissions. Therefore, we use the total population as a control variable.

Infrastructure (IF) is the area per capita of urban road areas. Zhang [8] believed that good infrastructure can not only bring a broad market but also enhance inter-regional communication. And, convenient transportation is conducive to attracting talents, capital, and other production factors. The influx of production factors and the expansion of the market have jointly promoted the transformation of the regional industrial structure and changed the regional carbon emission pattern. Therefore, we use the infrastructure as a control variable.

Research and development intensity (*R&D*) is the ratio of the city’s scientific research investment to the regional GDP. Santen, Webster [34] and Wang, Chen [11] believed that there is a significant positive correlation between R&D intensity and economic growth, meaning that a moderate increase in R&D investment will help to rapidly improve the level of technological innovation and accelerate the low-carbon economic transition. Therefore, we use the R&D intensity as a control variable.

Economic development level (*EL*) is the city’s per capita GDP. Xu, He [37] used the LMDI method to analyze the influencing factors of China’s fossil energy carbon emissions from 1995 to 2011 and believed that economic development level is an important factor affecting carbon emissions. Therefore, we use the economic development level intensity as a control variable.

For the consideration of heteroscedasticity, this article takes the logarithm of total population (*lnPOP*), economic development level (*lnEL*), and infrastructure (*lnIF*). The descriptive statistics of the main variables are in Table 1.

**Table 1:** Descriptive statistics.

VARIABLES	Obs	Mean	Std. Dev.	Min	Max
***lnCP***	1,890	2.9035	0.9163	-1.047867	12.8456
***lnLCI***	1,890	3.8898	1.6518	0	9.5273
***IS***	1,890	1.4756	0.6768	0.052	7.21017
***FDI***	1,890	0.01756	0.0162	0	0.0932
***POP***	1,890	5.8959	0.6972	2.9226	7.2794
***EL***	1,890	10.0176	0.5873	8.1022	13.4257
***R&D***	1,890	-6.3815	0.6798	-8.9619	-2.763
***IF***	1,890	2.3045	0.5935	-1.1712	4.6856

### Difference-in-difference model

The DID approach can avoid possible endogenous problems, and is used by numerous studies that evaluate the carbon emission trading pilot policy. Thus, we adopted the followed DID model to measure the influence of the LCCP policy on China’s low-carbon economic transition:

$$\mathrm{lnC}P_{it}=\alpha_{0}+\alpha_{1}pilot_{i}\times post_{t}+\alpha_{2}X_{it}+\mu_{i}+\gamma_{t}+\varepsilon_{it}$$ (1)

Subscripts *i* and *t* represent the province and the year, respectively. In Eq (1) lnCP_it_ represents the carbon productivity. The dummy variable *pilot* is the regions of policy implementation and is assigned a value of 1 or 0 for the region implementing the policy and non-implementing the policy in 2012, respectively. The dummy variable *post* is the period of policy implementation and is assigned a value of 1 or 0 for the period after (t ≥2012) and before (t <2012) the policy implementation, respectively. X represents the control variables. μ_*i*_ and γ_*t*_ represent fixed effect in control province and fixed effect in control time, respectively. ε_*it*_ is the residual. α_1_ represents the influence of the LCCP policy on the low-carbon economic transition.

### Robustness test

The prerequisite for using the DID approach is that if the pilot policy is not implemented, the trend of the export technical sophistication in pilot regions and non-pilot regions should be parallel. To ensure the reliability of the DID model, we use Eq (2) to perform a parallel trend test.


$$\mathrm{lnC}P_{it}=\beta_{0}+\Sigma_{t=2008}^{2011}\beta_{t}pilot_{i}\times d_{t}+\beta_{1}X_{it}+\mu_{i}+\gamma_{t}+\varepsilon_{it}$$ (2)

In Eq (2), dummy variable dt represents year (t = 2008, 2009, 2010, 2011). For example, if the year is 2008, then d_2008_ takes on value 1, and d_2009_, …, d_2011_ all take on 0. In Eq (2), our attention is focused on the coefficient β_*t*_. Theoretically, the DID model satisfies the parallel trend hypothesis test, when β_*2008*_, β_*2009*_, β_*2010*_, and β_*2011*_ are not significant.

### Mediation effect model

Porter Hypothesis holds that proper environmental regulations can urge enterprises to develop technological innovation, which lowers the production costs and ultimately benefit foreign trade [22]. The LCCP policy used administrative methods and tax incentives to stimulate enterprises to develop low-carbon technologies [23]. Additionally, the policy adopted various ways to subsidize related enterprises, such as low-carbon development funds, investment subsidies, loan interest discounts, direct rewards, and project management fee subsidies, to expand their R&D expenditures on low-carbon technology. Technological innovations lead to higher carbon productivity, which not only compensates for the compliance cost but also makes the enterprises generate fewer carbon emissions than those not investing in low-carbon technologies [24].

We extract low-carbon technological innovation as a potential mediation variable from a theoretical analysis mentioned above to explore the influence channel that the LCCP policy promotes China’s low-carbon economic transition. To test this influence channel empirically, we established mediation effect models, namely Eqs (3)–(5).


$$\mathrm{lnC}P_{it}=\alpha_{0}+\alpha_{1}pilot_{i}\times post_{t}+\alpha_{2}X_{it}+\mu_{i}+\gamma_{t}+\varepsilon_{it}$$ (3)

$$\mathrm{lnLC}I_{it}=\alpha_{0}+\beta_{1}pilot_{i}\times post_{t}+\beta_{2}X_{it}+\mu_{i}+\gamma_{t}+\varepsilon_{it}$$ (4)

$$\mathrm{lnC}P_{it}=\alpha_{0}+\lambda_{1}pilot_{i}\times post_{t}+\lambda_{2}\mathrm{lnLC}I_{it}+\lambda_{3}X_{it}+\mu_{i}+\gamma_{t}+\varepsilon_{it}$$ (5)

Eq (3) is a benchmark DID model. In Eq (4), *lnLCP*_*it*_ as the explained variable represents the low-carbon innovation of provinces *i* in time *t*. And Eq (5) is to add *lnLCI*_*it*_ to Eq (3). The mediation effect is tested by stepwise regression.

In the first place, we discuss regression coefficient α_1_ in Eq (3). If α_1_ is not significant, the causal relationship between the LCCP policy and low-carbon economic transition is weak. So, the mediation effect test ends. But if α_1_ is significant, we continue to construct Eq (4) to discuss whether the LCCP policy affects the low-carbon innovation. If *β*_*1*_ is not significant, the causal relationship between the policy and the low-carbon innovation is weak. So, the mediation effect test ends. But if *β*_*1*_ is significant, we continue to construct Eq (5) to discuss whether there is a mediation effect on low-carbon innovation. In Eq (5), if the regression coefficients both λ_1_ and λ_2_ are significant and λ_1_ is closer to 0 than α_1_, the low-carbon innovation is a mediation variable for the LCCP policy to influence the low-carbon transition. And the mediation effect is partial. If the regression coefficient λ_1_ is not significant, but the regression coefficient λ_2_ is significant, the low-carbon innovation is also a mediation variable for the LCCP policy to influence low-carbon transition. In this case, the mediation effect is full. If neither of them is significant, low-carbon innovation is not a mediation variable for the LCCP policy to influence the low-carbon transition.

### Implementation tools analysis

As mentioned above, the local governments use three types of policy tools to construct low-carbon cities, including command-mandatory tools, market-economic tools, and voluntary tools. We intend to analyze whether the policy implementation tools generate a heterogeneous influence on the low-carbon economic transition in the pilot areas. And Eq (6) is constructed to fulfill this analysis.


$$\mathrm{lnC}P_{it}=\alpha_{0}+\alpha_{1}{\mathrm{CMT}}_{it}+\alpha_{2}\mathrm{ME}T_{it}+\alpha_{3}\mathrm{VL}T_{it}+\alpha_{4}X_{it}+\mu_{i}+\gamma_{t}+\varepsilon_{it}$$ (6)

In Eq (6), *CMT*_*it*_ represents the command-mandatory tools and is the ratio of the number of command-mandatory tools to the total number of tools. *MET*_*it*_ indicates the market-economic tools and is the ratio of the number of market-economic tools to the total number of tools; VLT_*it*_ represents the voluntary tools and is the ratio of the number of voluntary tools to the total number of tools. It needs to be underlined that the samples used in this analysis are only from the pilot regions.

## Empirical test and analysis

### Benchmark regression results

Based on Eq (1), we empirically analyzed the influence of low-carbon city pilot policy on China’s low-carbon economic transition. The results are in Table 2. The regression results in column (1) do not add any control variables, and then control variables are gradually added from columns (2)-(7). The empirical results found that the DID regression coefficients, namely *pilot×post*, from column (1)-(7) are all significantly negative, meaning that the LCCP policy has a significant negative influence on China’s low-carbon economic transition. The pilot policy has not promoted but inhibited the low-carbon economic transition. The regression results are not consistent with Hypothesis 1. This may be because the LCCP policy is weakly binding. At the national level, the NDRC did not set specific policy targets, such as the time of carbon emissions peak and the emission standards in different industries. Therefore, local governments in the pilot regions make low-carbon efforts based on their conditions and capabilities. Compared with other environmental regulations or policies, the policy is short of a clear definition, effective evaluation system, and specific goals, which leads to distortion of resource allocation and loss of efficiency. Therefore, the policy cannot promote China’s low-carbon economic transition. The results are consistent with the research of Sinn [14], [12], and [13]. Therefore, the LCCP policy needs to be optimized.

**Table 2:** Regression results of the LCCP policy on China’s low carbon economic transformation.

	(1)	(2)	(3)	(4)	(5)	(6)	(7)
VARIABLES	lnCP	lnCP	lnCP	lnCP	lnCP	lnCP	lnCP
**pilot×post**	-0.177**	-0.174**	-0.160**	-0.160**	-0.158**	-0.161**	-0.147**
	(0.0752)	(0.0748)	(0.0753)	(0.0754)	(0.0754)	(0.0752)	(0.0751)
**IS**		0.213***	0.213***	0.213***	0.202***	0.212***	0.201***
		(0.0451)	(0.0451)	(0.0451)	(0.0465)	(0.0465)	(0.0465)
**FDI**			2.233	2.238	2.230	2.074	1.923
			(1.601)	(1.601)	(1.601)	(1.599)	(1.594)
**POP**				0.0590	0.0709	0.179	0.321
				(0.286)	(0.286)	(0.288)	(0.289)
**EL**					0.0533	0.0661	0.0666
						(0.0578)	(0.0576)
**RD**						-0.120***	-0.132***
						(0.0407)	(0.0407)
**IF**							0.180***
							(0.0518)
**City FE**	YES	YES	YES	YES	YES	YES	YES
**Year FE**	YES	YES	YES	YES	YES	YES	YES
**Observations**	1890	1890	1890	1890	1890	1890	1890
**R-squared**	0.718	0.722	0.722	0.722	0.722	0.724	0.726

Note: The robust standard errors are shown in parentheses

***, **, and * represent significance at the 1%, 5%, and 10% levels, respectively. Limited to space, the following tables are the same.

### Robustness test

To ensure the reliability of the empirical results, this article conducted three robustness tests. The results are in Table 3. And all of them confirmed that the LCCP policy has a significant inhibitory effect on China’s low-carbon economic transition.

**Table 3:** Results of robustness test.

	(1)	(2)	(3)	(4)	(5)
VARIABLES	lnCP	lnCP	lnCP	lnCP	lnCP
	parallel trend	pilot×t_2009_	pilot×t_2010_	pilot×t_2011_	provincial time trend
**pilot×post**		0.559	0.289	0.114	-0.128*
		(0.398)	(0.198)	(0.137)	(0.0753)
**pilot×t**_**2008**_	-0.319				
	(0.214)				
**pilot×t**_**2009**_	0.262				
	(0.276)				
**pilot×t**_**2010**_	0.362				
	(0.281)				
**pilot×t**_**2011**_	0.274				
	(0.181)				
**control variables**	YES	YES	YES	YES	YES
**City FE**	YES	YES	YES	YES	YES
**Year FE**	YES	YES	YES	YES	YES
**Observations**	1890	840	840	840	1890
**R-squared**	0.73	0.847	0.843	0.84	0.726

Firstly, we used Eq (2) to perform a parallel trend hypothesis test. Column (1) expresses the regression results of the parallel trend hypothesis test. Before the policy implementation, the regression coefficients of *pilot×post*, namely *pilot×t2008*, *pilot×t2009*, *pilot×t2010*, and *pilot×t2011*, are not significant, which indicates that there was no significant difference in China’s low-carbon economic transition between the experimental group and the control group. In other words, the LCCP policy conforms to the parallel trend hypothesis test.

Secondly, we performed a placebo test. The test is to separately assume that the implementation year of the LCCP policy is 2009, 2010, and 2011, and remove samples in 2012 and later. Base on Eq (1), the placebo test results are in Table 3. Columns (2)-(4) are not significant, so the regression results about the effect of the LCCP policy on the low-carbon economic transition are robust.

At last, we added the provincial time trend, namely the multiplicative interaction term of *pilot* and *year*, to Eq (1) as a new control variable. The regression results are shown in column (2). After adding the variable, the regression coefficient of *pilot*×*post* is still significantly positive, meaning that some of the time-varying provincial factors that may be omitted do not have a substantial influence on the above conclusions. And the results confirm the robustness of the benchmark regression results as well.

### Regional heterogeneity analysis

The above empirical analysis discussed the effect of the LCCP policy on China’s low-carbon economic transition from a national perspective. However, China is a developing country with unbalanced regional development, and the efficiency of policy implementation is frequently heterogeneous in regions. So, it is essential to analyze the regional heterogeneity of the policy implementation.

Referring to the classification standards of the National Bureau of Statistics, this article divides China into three regions, namely the eastern region, central region, and western region. The east region includes Beijing Municipality, Hebei Province, Jiangsu Province, Shandong Province, Hainan Province, Shanghai Municipality, Zhejiang Province, Fujian Province, Tianjin Municipality, Guangdong Province, and Liaoning Province; The central region includes Shanxi Province, Hunan Province, Jiangxi Province, Hubei Province, Jilin Province, Heilongjiang Province, Henan Province, Anhui Province, Inner Mongolia Province, and Guangxi Province; The west region includes Chongqing Municipality, Qinghai Province, Gansu Province, Guizhou Province, Ningxia Province, Shanxi Province, Yunnan Province, Xinjiang Province, and Sichuan Province. The geographical location of the eastern region is excellent, and the region occupies a predominant management system and superior financial support. The central region is geographically connected with the coast in the east and inland in the west, and its economic level is in the middle level among the three regions. Unfortunately, economic development in the western region is disappointing, reflected in the large gap between the western region and the eastern region in resource endowments, infrastructures, and industrial structures. Therefore, the effects of the LCCP policy may be different among the three regions.

This paper further studied the policy implementation effects on low-carbon economic transition in regions based on Eq (1). The results are in Table 4. The regression coefficient of the core variable, namely *pilot×post*, is positive in the eastern region but negative in the western region and cannot pass the test at a 10% significance level in the central region. The results imply that the LCCP policy has an ascendant effect on the low-carbon economic transition in the eastern region but is unfavorable for the western region. So, what caused the same policy to generate opponent effects on the low-carbon economic transition in regions? To solve this puzzle, we analyzed the influence mechanism in the next section.

**Table 4:** Regional heterogeneity analysis.

	(1)	(2)	(3)
VARIABLES	eastern region	central region	western region
	**lnCP**	**lnCP**	**lnCP**
**pilot×post**	0.0503*	-0.0434	-0.411**
	(0.03)	(0.069)	(0.198)
**control variables**	YES	YES	YES
**City FE**	YES	YES	YES
**Year FE**	YES	YES	YES
**Observations**	567	783	540
**R-squared**	0.894	0.657	0.751

### Regional influence channel analysis

As mentioned above, the pilot policy demonstrated apparent heterogeneity among regions. Specifically, the pilot policy generates an adverse effect on the low-carbon economic transition in the ordinary central region and the underdeveloped western region but an advantageous effect in the prosperous eastern region. Based on Eqs (3) to (5), we used the stepwise regression method to explore the regional influence channel.

The regression results are in Table 5. the regression coefficients of “*pilot×post*” in columns (1)-(2) and the coefficient of *lnLCI* in column (3) are all significantly positive, indicating that low-carbon innovation is a mediation variable for the LCCP policy to enhance the low-carbon economic transition in the eastern region. And the mediation effect is partial. However, the regression coefficients of “*pilot×post*” in columns (4)-(7) cannot pass the test at a 10% significance level, meaning that the pilot policy does not have a significant influence on the low-carbon innovation and low-carbon economic transition in the central region and western region. Specifically, the empirical results show that in the eastern region the LCCP policy can stimulate low-carbon innovation with the help of innovation offset effects, and advanced low-carbon innovation benefits economic transition. However, the LCCP policy did not bring innovation compensation to the central region and western region, resulting in stagnation of the low-carbon economy in those regions. Therefore, whether the policy effectively stimulates technological innovations is the key to construct a low-carbon economy.

**Table 5:** Regional influence channel.

	(1)	(2)	(3)	(4)	(5)	(6)	(7)
VARIABLES	lnCP	lnLCI	lnCP	lnCP	lnLCI	lnCP	lnLCI
	eastern region			central region		western region	
**pilot×post**	0.0503*	0.143**	0.05*	-0.0434	0.162	-0.411**	-0.1811
	(0.03)	(0.0575)	(0.03)	(0.069)	(0.118)	(0.198)	(0.1148)
**lnLCI**			0.0045*				
			(0.0026)				
**Control variables**	YES	YES	YES	YES	YES	YES	YES
**City FE**	YES	YES	YES	YES	YES	YES	YES
**Year FE**	YES	YES	YES	YES	YES	YES	YES
**Observations**	567	567	567	783	783	540	540
**R-squared**	0.894	0.977	0.894	0.657	0.925	0.751	0.934

The performance of the policy is affected by the local industrial structures and technological innovation capability. Compared with other regions, industries in the eastern region are mainly capital-intensive and technology-intensive. Therefore, most enterprises in the eastern region naturally have a broad space for innovation, which cultivates their innovative sensitivity and shapes their innovative capabilities. Furthermore, Local governments in the eastern region value the talent and are determined to improve their working environment and welfare benefits. In brief, the industrial structures provide innovation incentives for local enterprises which employ multitudinous marvelous brains because of the government’s talent policies. Therefore, pilot cities in the eastern region can smoothly implement the LCCP policy and local enterprises benefit from it.

However, industries in the central region and western region are generally resource-intensive and labor-intensive, making enterprises in these regions lack sufficient capital investment, human capital, and advanced technology which are essential for low-carbon technological innovation. Insufficient innovation capabilities make some local enterprises pay exorbitant compliance costs by reducing production in the short term when the pilot policy is implemented. The enterprises failed to obtain compliance compensation through technological innovation, so in the central region and western region, the LCCP policy cannot serve the low-carbon economic transition.

### Performance of policy tools analysis

As mentioned above, local governments generally use three types of policy tools to establish low-carbon cities, including command-mandatory tools, market-economic tools, and voluntary tools. The performance of policy tools differs among pilot regions, so we intend to analyze whether the different types of policy tools generate a heterogeneous influence on the low-carbon economic transition in the pilot areas. And Eq (6) is constructed to fulfill this analysis. The regression results are in Table 6. The empirical results reveal that market-economic tools are valuable to improving the low-carbon economic transition in pilot areas, but command-mandatory tools and voluntary tools have failed.

**Table 6:** The impact of policy tools on the transformation of low carbon economy.

	(1)	(2)	(3)
VARIABLES	lnlci	lnlci	lnlci
**CMT**	-0.669		
	(0.619)		
**MET**		0.612*	
		(0.372)	
**VLT**			-0.353
			(0.436)
**Control variables**	YES	YES	YES
**City FE**	YES	YES	YES
**Year FE**	YES	YES	YES
**Observations**	120	120	120
**R-squared**	0.972	0.972	0.972

From our perspective, the strict and inflexible command-mandatory tools often overestimate the corporate capability of pollution control. Under the pressure of administrative commands, enterprises had to choose alternatives to reduce energy consumption, leading to higher costs and a decline in their comparative advantages and profitability. Additionally, strict environmental policies forced enterprises to spend time, funds, and manpower on management, which reduced their willingness to develop low-carbon technological innovation [27]. As a result, command-mandatory tools failed to evoke the low-carbon economic transition in pilot areas. However, flexible market-economic tools not only effectively stimulate enterprises to innovate low-carbon technology but bring innovation compensation to participating enterprises. Therefore, market-economic tools are advantageous to reduce carbon emissions and beneficial to accelerate the low-carbon economic transition.

## Conclusions, recommendations, and limitations

Based on the data of 210 prefecture-level cities in China, this paper empirically analyzed the effect of the LCCP policy on China’s low-carbon economic transition by using DID analysis. The main conclusions are as follows. (1) The LCCP policy cannot generate the Porter effect and inhibits China’s low-carbon economic transition in general. However, the LCCP policy has regional heterogeneity. Specifically, the LCCP policy encourages the low-carbon economic transition in the eastern region but hinders it in the central region and western region. (2) The low-carbon technological innovation is a mediation variable for the LCCP policy to influence low-carbon economic transition. And the innovation offset effects have been generated in the eastern region but not in the central region and western region. (3) Market-economic tools are valuable to improving the low-carbon economic transition in pilot areas, but command-mandatory tools and voluntary tools have failed.

Based on these conclusions, we made several recommendations for improvement. (1) Because of the institutional defects the LCCP policy could not play the expected role. The policymakers need to formulate clearer low-carbon city development goals as well as an effective evaluation system and pay attention to specific programs to encourage low-carbon technological innovation. (2) The government might support enterprises to work with scientific research institutions and universities to innovate technologies. In the meantime, the NDRC and local governments could provide corresponding innovation subsidies for participating enterprises to stimulate them to develop low-carbon technological innovations. (3) Given different levels of development in cities, policy fairness should be emphasized by the NDRC When policymakers formulate low-carbon policies. (4) Market-economic tools can effectively improve the low-carbon economic transition in pilot areas, so market-economic tools, such as carbon emissions trading, are predominant choices for local governments to establish a green city.

The main contributions of this article are to empirically analyze the influence of the LCCP policy on China’s economic transition, provide direct empirical evidence for optimizing the policy, and provide practical experience for the world, especially developing countries and regions. The limitation of this article is that the LCCP policy may affect the low-carbon economic transition through other channels, such as the efficiency of resource allocation, but this article only analyzes one of them, low-carbon technological innovation. We intend to research the efficiency of resource allocation and other influence channels in follow-up research.

## Supporting information

S1 Data (XLS)
